# News and Miscellany

**Published:** 1902-07

**Authors:** 


### News and Miscellany.

---

**This number** begins the eighteenth year of "The Famous Red Back."

---

**Dr. Jos. Gilbert,** of Austin, has succeeded Dr. L. D. Hill as surgeon to the Confederate Home, at Austin.

---

**Dr. T. T. Jackson,** of San Antonio, has been appointed Division Surgeon of the Southern Pacific Railroad, vice Dr. Amos Graves, Sr., who has held the position many years.

---

**The new law** regulating the practice of medicine in Texas can be had in pamphlet form by enclosing 20 cents (stamps or silver) to this office. TEXAS MEDICAL JOURNAL,
Austin, Texas.

---

**The West Texas Medical Association** held an interesting meeting at San Antonio June 26. The next meeting will be at New Braunfels, July 31 (inst.), upon invitation of the President, Dr. A. Garwood, of that city.

---

**Dr. M. M. Smith,** our esteemed contemporary of the *Texas Medical News*, and Secretary State Board of Medical Examiners, is in New York attending the famous "Rebel School"—"Wyeth's School", as the Southern doctors all call ye N. Y. Polyclinic. Dr. Smith was accompanied by his beautiful and accomplished wife and his two splendid boys, Girard and Matthew M. Lucky fellow, Smith is, and deservedly so.

---

**Dr. J. S. Lankford,** of San Antonio, was recently elected a member of the board of school trustees of that city. Dr. Lankford has given much attention to the subject of education and to that of school hygiene, and the city is to be congratulated upon his election. He will be a zealous advocate of reform in school hygiene and will advocate vaccination, ventilation, shorter hours of study and more out-of-door exercise, etc.

**The Journal** is gratified to note that Dr. H. A. West, Secretary Texas State Medical Association, who represented the profession of Texas at the Saratoga meeting of the American Medical Association (June 3-9 ult.) was appointed on three important committees; towit: Public Health, Transportation, Business. Dr. West did all in his power to secure the 1903 meeting for San Antonio. So did Dr. Paschal, who appeared before the nominating committee and personally invited the Association in the name of the Governor, as well as in behalf of the medical profession. But the fear of inadequate hotel accommodations at San Antonio caused the Association to decline the invitation, and New Orleans was selected instead. Meeting, May, 1903. Dr. Frank Billings of Chicago was elected president.

---

**A Bacteriologic Tragedy.**—A gay bacillus, to gain him glory, once gave a ball in a laboratory. The fete took place on a cover glass, where vulgar germs could not harass. None but the cultured were invited (for microbe cliques are well united), and tightly closed the ball-room doors to all the germs containing spores. The Staphylococci first arrived—to stand in groups they all contrived; the Streptococci took great pains to set themselves in graceful chains; while somewhat late, and two by two, the Diplococci came in view. Pneumococci, stern and haughty, declared the Gonococci naughty, and would not care to stay at all if they were present at the ball. The ball began, the mirth ran high, with not one thought of danger nigh. Each germ enjoyed himself that night, with never a fear of Phagocyte. 'Twas getting late (and some were "loaded"), when a jar of formalin exploded, and drenched the happy dancing mass who swarmed the fatal cover glass. * * * Not one survived, but perished all, at this bacteriologic ball.—J. L. Hagadorn, M. D., in *Southern Cal. Practitioner.*

---

### The American Congress of Tuberculosis.

---

Atlanta, Ga., June 14, 1902.

*Editor Texas Medical Journal, Austin Texas.*

Dear Sir: At a meeting of the American Congress of Tuberculosis held in New York June 3rd, 4th and 5th, a reorganization was effected and the following officers elected for the ensuing year:

Honorary President, Dr. Henry D. Holton, Brattleboro, Vt.; President, Dr. Daniel Lewis, New York, N. Y.; First Vice President, Dr. J. A. Egan, Illinois; Second Vice President, Dr. Frank Paschal, San Antonio, Texas; Third Vice President, Dr. E. J. Barrack, Toronto, Canada; Fourth Vice President, Dr. J. A. Watson, Concord, N. H.; Fifth Vice President, Dr. Romola, Gautemala; Secretary, Dr. George Brown, Atlanta, Ga.; Treasurer, Dr. P. H. Byrce, Toronto, Canada.

The suggestion to hold a World's Congress of Tuberculosis in St. Louis in 1904 met with approval and steps are being taken to advertise this fact and secure the aid of medical journals, societies, physicians, and scientists in making this movement a grand success. I would appreciate it very much if you would interest yourself with us in this movement and give us your aid and advice.

Very respectfully,

GEORGE BROWN, Secretary.

---

YOAKUM, TEXAS, May 28, 1902.

*Dr. F. E. Daniel, Austin, Texas.*

DEAR SIR: Enclosed please find M. O. to renew my subscription to the "Red Back." I think a journal that is as devoted to the medical profession of the State of Texas as the "Red Back" is, and one that is doing and has done the amount of good for the profession that is done by the "Red Back" should have the support of EVERY PHYSICIAN *in the State.* Keep my JOURNAL coming.

Very truly,

J. S. YOUNGKIN, M. D.

That's what they *all* say.—DANIEL.

---

### Prevention of Consumption in Texas.

(REPORT OF SPECIAL COMMITTEE.)

---

DALLAS, TEXAS, May 6, 1902.

*To the President and Members of the Texas State Medical Association.*

GENTLEMEN: The undersigned committee, appointed at the last annual meeting of the Association to report upon some plan looking toward uniformity of action for the prevention of tuberculosis in Texas, beg to report as follows:

In the opinion of your committee efforts to prevent the spread of tuberculosis should be directed (a) toward the public control of the disease, and (b) toward securing the co-operation of every practicing physician in instructing tuberculous patients and their friends as to the dangers of the disease and how to dispose of the sputum without endangering the health of others.

A.—THE PUBLIC CONTROL OF TUBERCULOSIS BY BOARDS OF HEALTH.

1. It is recommended that all local boards of health declare tuberculosis to be a communicable disease; that they establish a system of registration and require all physicians to notify the health office of the existence of every case of tuberculosis within one week after the patient comes under observation.

2. That so far as possible boards of health distribute among tuberculous patients and their friends circulars or pamphlets telling in the plainest language how tuberculosis spreads and how it may be prevented (when requested, this might be done by the visit of a sanitary inspector).

3. That every room occupied by a tuberculous patient be thoroughly disinfected after it is vacated by either the death or the removal of the patient, and that a record of such disinfection be kept.

4. We believe that the enforcement of ordinances prohibiting spitting in public buildings, in cars, on pavements, etc., is of distinct educational value in calling the attention of people to the dangers which may arise from promiscuous spitting.

5. It is recommended that this Association endeavor to secure the establishment of a State Bacteriological Laboratory with a salaried bacteriologist, so that sputum examinations may be made free of charge, thus making it possible to establish a certain diagnosis early in the course of the disease. A laboratory of this kind wouid also be of great advantage in making cultures from all doubtful cases of diphtheria and determining how long such cases should be isolated.

6. In our opinion the weight of evidence is in favor of the unity of tuberculosis, and we recommend that the inspection of dairy herds supplying milk to cities be continued.

7. That whenever practicable it is very desirable to establish sanatoria in suitable localities for the care of tuberculous patients.

B.—METHODS RECOMMENDED IN DEALING WITH INDIVIDUAL TUBERCULOUS CASES.

1. It is recommended that attending physicians be perfectly candid in informing the patient, or at least the friends of the patient, of the nature of the disease, its dangers and the methods of prevention. To conceal the diagnosis is an injustice both to the patient and his friends, as the former is not as careful of himself as he would be if he knew the truth, thus lowering the chances of his recovery, and at the same time is careless about expectorating, and may reinfect himself and infect those about him.

2. That attending physicians be urged to fully instruct tuberculous patients in the use of sputum cups, sputum pocket flasks, or Japanese paper napkins, so that the patient may know how to dispose of the sputum under any circumstances, whether at home or not; that the dangers of promiscuous expectoration, the use of cuspidors and of handkerchiefs be carefully pointed out.

3. By the distribution of printed pamphlets or circulars, giving explicit directions as to the above, the private physician will usually accomplish more than by verbal instructions alone.

4. That private physicians be requested to co-operate with the board of health in every way, and especially in reporting every case of tuberculosis who, through ignorance or carelessness, disregards instructions as to the disposal of sputum.

Respectfully submitted by

W. S. Carter, M. D,
Allen J. Smith, M. D.,
Frank Paschal, M. D.,
Walter Shropshire, M. D.,
Committee.

